# Environmentally benign carbon nano dots as luminescence probe for quantification of palladium (II) chloride impurity in naproxen

**DOI:** 10.1186/s13065-025-01600-4

**Published:** 2025-08-06

**Authors:** Miranda F. Kamal, Rana M. Moustafa, Wael Talaat, Rasha M. Youssef

**Affiliations:** 1 Department of pharmaceutical analytical chemistry, Faculty of pharmacy, Damanhour University, Beheira, Egypt; 2 PharmD Program, Egypt-Japan University of Science and Technology (E- JUST), New Borg El-Arab City, Alexandria Egypt; 3 Department of pharmaceutical analytical chemistry, Faculty of pharmacy, Alexandria University, Alexandria, Egypt

**Keywords:** Naproxen, Indirect spectrophotometry, Fluorescence quenching, Validation

## Abstract

Green shades of “The 2030 Global Green Agenda” have directed the analysts into a peaceful relationship with surrounding environment. Natural resources, clean energy, green solvents are in commitment with Earth’s sustainability. Hazardous inorganic chemical reagents are in gradual replacement by nanoscale reactive species of green origins. In this sense, the present study utilizes nano Carbon Dots, synthesized from garlic peels as natural precursor, with a modified surface activity “multiple active chromophores”. The latter act as potent fluorogenic scavengers for optical nano sensing of Palladium traces in the anti-inflammatory Naproxen drug substance. Palladium traces are found in Naproxen bulk as synthesis-related impurity. Higher ingested levels increase the risk of debilitating diseases in the human body. Stoichiometric reaction between Palladium traces and the dots’ active surfaces was quantified indirectly along the linear spectral diminish of carbon dots luminescence. The proposed methods involve quantitative fluorescence quenching of carbon dots at 432 nm upon excitation at 375 nm and absorption peak at 250 nm upon addition of studied palladium chloride. The obtained results were in good agreement according to the Stern-Volmer equation along the linear range of 0.0088–0.8870 µgmL^-1^ palladium chloride. The developed procedures were validated as per ICH guidelines.

**Supplementary Information:**

The online version contains supplementary material available at 10.1186/s13065-025-01600-4.

## Graphical Abstract

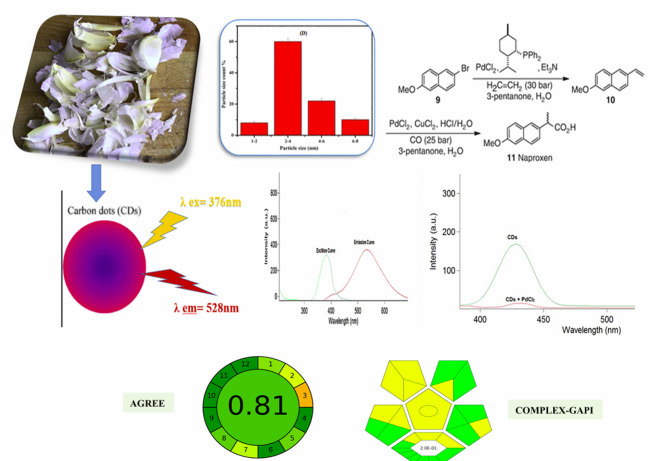

## Introduction

Carbon dots (CDs) are recently considered a novel type of zero-dimensional fluorescent nano masses with active surface capabilities. Due to their unique optical properties, CDs can react with and sense biomolecules such as enzymes, nucleic acids and trace metal ions [1]. One of the most critical features of these CDs, along with their potent luminescent nature, is their hydrophilicity, which allows their use in bioimaging applications. Also, they are nowadays recognized as a prospective material due to their great photostability, favorable biocompatibility, and low toxicity. In this sense, the scientific community’s interest in these nanomaterials has grown exponentially [2, 3]. Recently, we recommended a green sensible strategy for the synthesis of fluorescent CDs [4] from garlic peels as natural progenitor as shown by Gaber et al. [5]. In the Supplementary file, there are explanations of the synthesis and characterizations of the nanodots utilized.

**Scheme 1:**
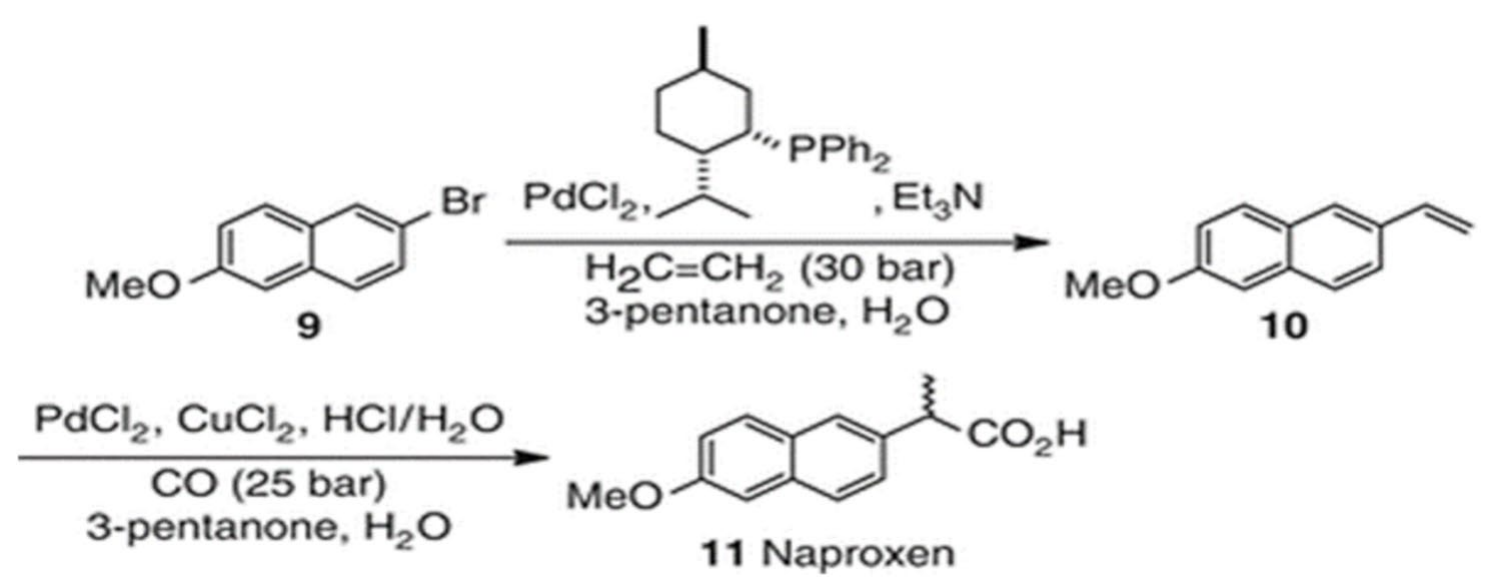
Naproxen Synthesis via Heck reaction followed by a hydroxyl carbonylation [8]

Palladium (Pd) is a rare transition noble metal that is a member of the Pt group and is crucial for many chemical and biological processes. Palladium has many applications such as dental crown, electronics, jewelry and medical instruments [6]. Additionally, palladium II chloride (PdCl_2_) is commonly used as a catalytic agent in one of the steps during synthesis of Naproxen (NPRX) via the Heck reaction of 2-bromo-6-methoxy-naphthalene on ethylene, followed by carbonylation of the product (Scheme 1) [7, 8]. The trace amount of Palladium remaining in the final product, which is not desirable as elemental impurity is rarely harmful to human health at low concentrations, but in some case can lead to a wide spectrum of debilitating diseases. So the contamination of Pd^2+^ persisted in reaction products needs further purifications. The ICH guidelines (Q3D) for Palladium which comes under class 2 elements where these elements are toxic to a greater or smaller extent list the daily permitted exposure at 100 mg /day for oral dosing and 10 mg /day for IV dosing [9].

The spectrophotometric detection of Pd^2+^ as inorganic impurity in drug substances by complexing with specific reagent has already been reported [9]. Moreover, there are different analytical techniques reported for determination of Pd^2+^ by complexing with various reagents [10–14]. These existing methods have numerous demerits such as low selectivity, limited range of pH as well as temperature control. Therefore, it is an urge to search for new selective, sensitive, available reagents which can be used in routine analysis. Herein, Carbon dots based fluorescent assays are known to overcome most of these drawbacks and are desirable candidates for use in determination of PdCl_2_. Sustainable carbon dots, i.e. green dots of natural precursors have proven up to 30% quantum yield along with reproducible assay results [15].

Literature reveals no reported method for spectrophotometric estimation of Palladium content in NPRX by using the luminescent carbon dots reagent. In this research, an environmentally friendly and effectual probe proposed by CDs is suggested to optimize and validate indirect photometric and fluorimetric determination of PdCl_2_ traces as synthesis related impurity in NPRX pharmaceutical formulation.

## Materials and methods

### Chemicals and reagents

Palladium (II) chloride pure standards were purchased from Sigma Aldrich (GERMANY). Naprofen^®^ tablets were purchased from Nile Pharmaceutical Co. All reagents and solvents used were of Analytical Grade. Concentrated Hydrochloric acid was purchased from El-Nasr for pharmaceutical and chemical industrial Co. (Egypt). Deionized water was obtained from Science Park Unit, Faculty of Pharmacy, Alexandria University. All solutions were prepared in deionized water.

### Instrumentation

All absorbance measurements were carried out with a Shimadzu UV Spectrophotometer (UV-1800) with 1.0 cm quartz cuvettes. A pH meter (HANNA, Portugal) was used for all pH measurements. Materials were weighed using an Axis analytical balance.

The fluorescence spectra were recorded by Cary Eclipse Fluorescence Spectrophotometer manufactured by Agilent technology containing a Xenon flash lamp, lamp pulse width at half peak height ~ 2 µs, peak power equivalent to 75 kW, Czerny-Turner monochromators with 12.5 cm focal width.

### Preparation and characterization of cds

The source of CDs garlic peels is dry-whitish outer layers of Egyptian garlic bulb that is sold in Egyptian market. Egyptian garlic is classified as Allium sativum, are small bulbs with numerous cloves found on a plant that can grow up to sixty centimeters in height and belongs to the Amaryllidaceae family, also known as a silverskin variety. It is cultivated in upper Egypt and sold in local market. The supplementary file describes the synthesis and meticulous characterization of used CDs.

### Preparation of standard stock solution of cds

CDs powder of 0.010 g, exactly weighed, was put into a 25-mL volumetric flask. It was made up to volume with the same solvent after being dissolved in 20 mL of deionized water. Every day, a fresh stock solution of CDs was made.

### Preparation of standard stock solution of PdCl_2_

Palladium (II) chloride, exactly weighed at 0.0178 g, was poured into a 50-mL volumetric flask, and then 0.5 mL of concentrated hydrochloric acid was added. It was solubilized in boiling water and then made up to volume using the same solvent. Every day, a fresh stock solution of PdCl_2_ was made.

### Construction of calibration curves

#### For spectrophotometric measurement of PdCl_2_

An exact volume of 1 mL of CDs stock solution was added into a 10 mL volumetric flask. To obtain the volume to mark, deionized water was used, resulting in a final concentration of 40 µg/mL. This prepared solution was examined. Separately, a set of 10-mL flasks was filled with a volume of 1 mL of the standard stock solution of CDs in each. To achieve the concentration range shown in Table 1, sequential aliquots of standard PdCl_2_ solution were added. Deionized water was added to the flasks after they had been well mixed. At 250 nm (λmax), the absorbance difference (A) was recorded for each concentration. Linearity was constructed between calculated ΔA and the corresponding PdCl_2_ concentrations.

#### For fluorimetric measurement of PdCl_2_

By micro pipping 0.1 mL of the standard stock CDs solution into a 10-mL volumetric flask, further dilution was accomplished. For a final concentration of 4 µg/mL, deionized water was added to the mark. The prepared solution’s emission fluorescence, F_o_, was measured at λem 432 nm following its excitation at λex 375 nm. Comparable to this, accurate amounts of 0.1 mL were quantitatively transferred into a set of 10-mL flasks from standard stock CDs solution. PdCl_2_ was separately added in sequential aliquots, and the marks were made with deionized water. Table 1 lists the final concentration ranges for fluorimetric measurements of PdCl_2_. Both the CDs solution and each solution of PdCl_2_ with CDs had their relative fluorescence intensities, F, measured (λex = 375 nm, λem = 432 nm). For each concentration of PdCl_2_, independently calculated calibration curves for ΔF (F_o_ − F) and F-ratio (F_o_/F) were built.

### Sample preparation (naprofen^®^)

Ten tablets were finely powdered and thoroughly mixed after being exactly weighed. A 10-mL volumetric flask was filled with a portion of the tablet powder equivalent to 10 mg NPRX. For drug extraction, a volume of 5 mL deionized water was added to the powder and 0.5 mL of the standard stock solution of PdCl_2_ was added then the flask was sonicated for 30 min. The sample solution was diluted to mark and filtered using 0.45-µm Millipore membrane filter. In a 10 mL volumetric flask 1 mL of the CDs reagent was added along with filtrate that contain PdCl_2_ and the volume was adjusted to the mark with deionized water. After thoroughly mixing, let stand for 5 min. The absorbance of complex was measured at 250 nm and quenching efficiency at 432 nm, upon excitation at 375 nm instantly and at room temperature against the blank solution.

## Results and discussion

Garlic peels were used to synthesize luminescent CDs in an environmentally friendly way [4, 5]. They served as sensing probes for the quantitative trace level detection of PdCl_2_ in NPRX and were extensively characterized, as shown in Supplementary file. In this study to avoid any potential interferences in distilled water, CDs solution is built up using deionized water.

### UV-Spectrophotometric measurements

**Fig. 1:**
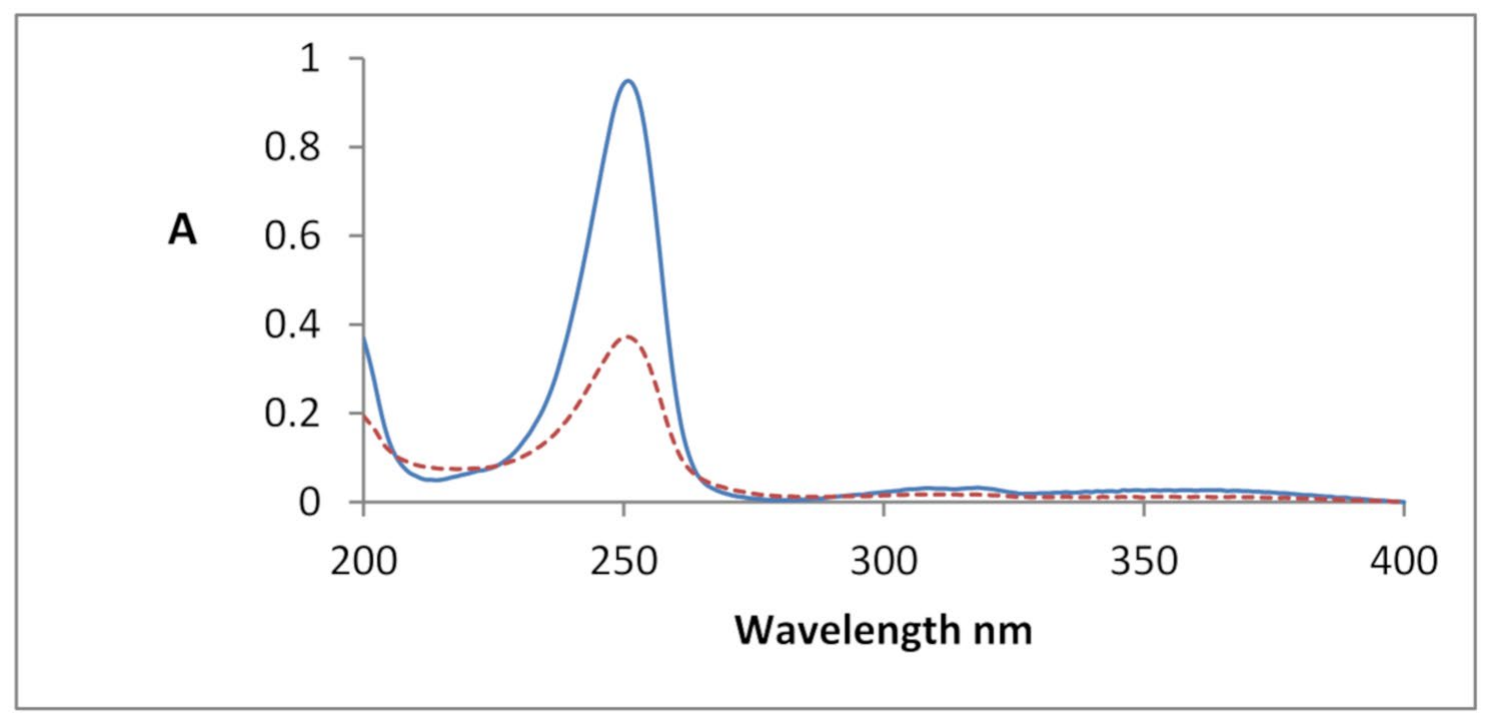
Absorption spectra of the complex formed between PdCl_2_ and CDs reagent; PdCl_2_ = 3.55 µg/mL and 40 µg/mL CDs reagent, pH = 7

An aqueous solution, 40 µg/mL, of CDs has a distinct absorption peak at λmax 250 nm. The chemical interaction between PdCl_2_ and CDs is observed by the decrease in the absorbance value of CDs, Absorbance difference ΔA (A_o_– A), is linearly related to PdCl_2_ concentrations. Where, A_o_ is the absorbance value of CDs solutionalone and A is the absorbance value of CDs with standard PdCl_2_ solution, both measured at λmax 250 nm Fig. 1.

### Fluorimetric measurements

**Fig. 2:**
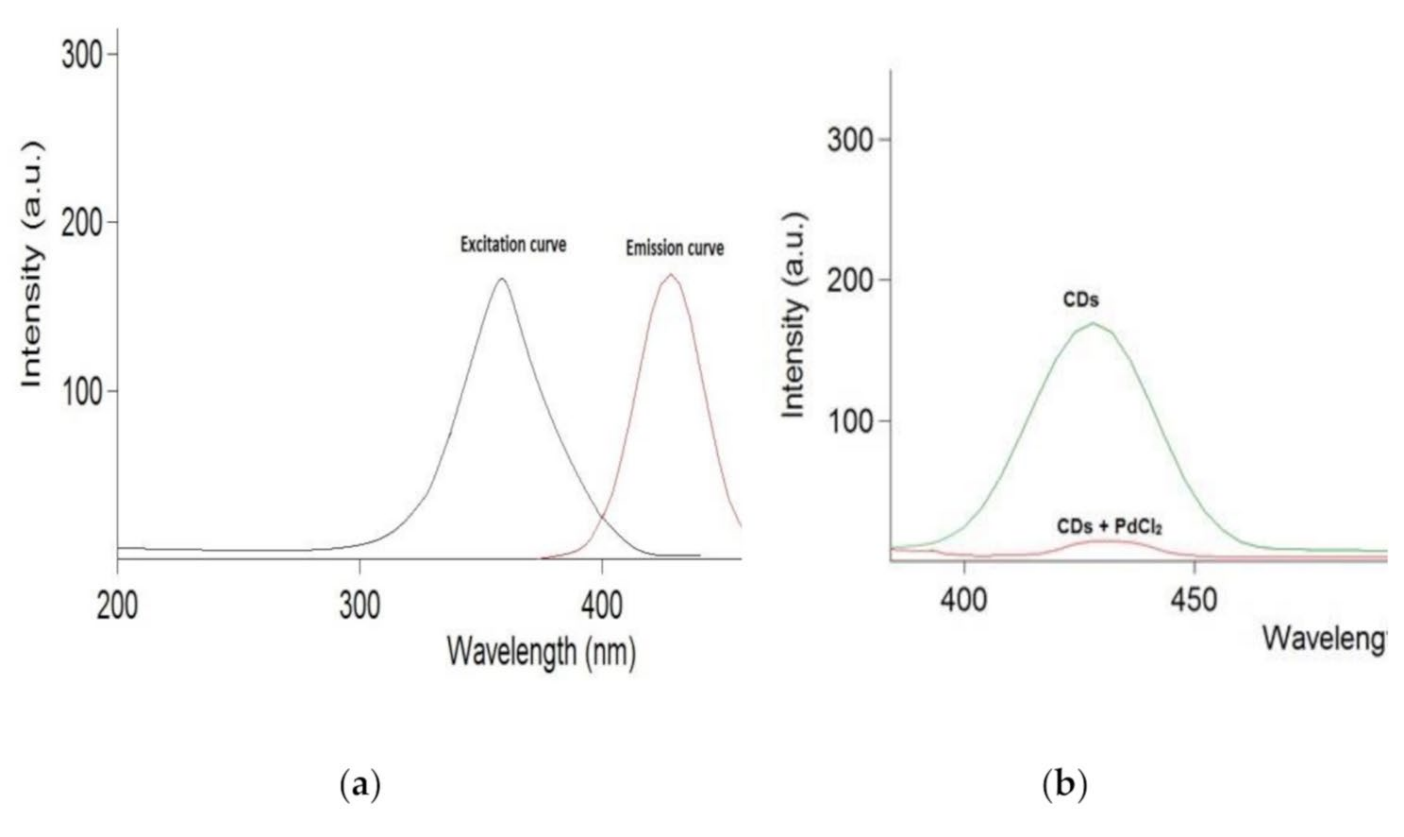
(**a**) Overlay for excitation and emission curves of CDs and (**b**) Quenching of 40 µg/mL CDs fluorescence emission in the presence of 3.55 µg/mL PdCl_2_, pH = 7

Comparable to this, upon excitation at λex 375 nm, aqueous CDs solution displays intrinsically powerful fluorimetric emission at λem 432 nm. When traces of PdCl_2_ standard solution are added, the fluorimetric emission of CDs solution is drastically decreased, Fig. 2. Generally, the active surfaces of CDs interact with PdCl_2_ ions may be attributed to effective chelation/coordination interactions between the metal ions and the amino, hydroxyl and carboxylic groups present in CDs [6, 16]. Furthermore, to explore the quenching mechanism of PdCl_2_ on CDs, we investigated the quenching behavior by way of the Stern–Volmer equation (Eq. 1) at 432 nm, upon excitation at 375 nm.

1 $${F_o}/F\, = \,{K_{sv}}\left[ Q \right]\, + \,1$$

Where F_o_ and F are the fluorescence intensities’ ratio in the absence and presence of PdCl_2_, respectively. If the emission magnitude in the absence of the quencher and then in the presence of incremental proportions of the quencher is measured, the resulting ratio (F_o_ /F) or the difference (ΔF = F_o_– F) of emission intensities is plotted as a function of quencher concentration [Q], with a commonly expected linear trend. The resulting graph (called a Stern–Volmer plot) will have an intercept of 1 and a slope called the Stern–Volmer constant, K_sv_. A Stern-Volmer plot is an intuitive method of determining quantitative information about fluorescence quenching when the nature of that quenching is perfectly well known.

In this study, to confirm the superiority of this method, a comparison was carried out between both calculated calibration curves; ΔF (F_o_ − F) and F-ratio (F_o_ /F) versus concentrations of PdCl_2_ separately. The results are presented in Table 1. We can clearly see that calculated F‐ratio (F_o_ /F) versus Pd conc has a better linear relationship with high correlation coefficient value (*R* > 0.999), small intercepts and high F-values rather than ΔF (F_o_ − F) one; which indicates high sensitivity of this method.

The K_sv_ depends on the temperature which is used to define whether the quenching mechanism is dynamic or static. In dynamic quenching, the quenching occurs via collision in excited state with fluorophore and quencher, so that as a temperature increases the collision and quenching increase. While, in static quenching, the quencher reacts with fluorophore to produce non-fluorophore complex in the ground state. So, the higher the temperature, the higher the complex dissociation and the decrease the quenching. In the present study, the K_sv_ was calculated at four temperatures (20, 30, 50 and 70 °C). It was found that the K_sv_ value decreased upon increase temperature which indicates occurrence of static quenching.

### Optimization of the reaction conditions

Noticeable features were tuned to select the optimum conditions necessary for the PdCl_2_ detection.

#### Effect of pH

**Fig. 3:**
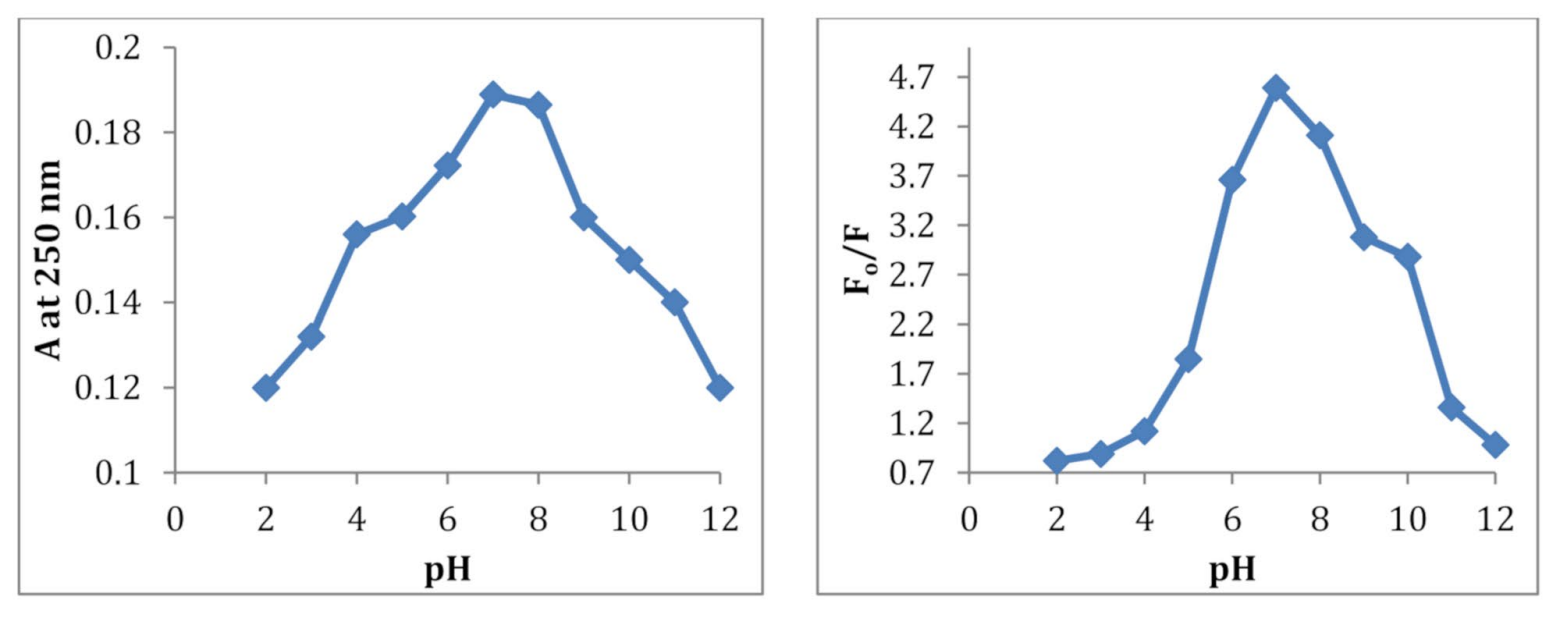
Effect of pH on the complex formed between PdCl_2_ and CDs reagent; PdCl_2_ = 3.55 µg/mL and 40 µg/mL CDs reagent

The impact of pH on each of the F_o_/F value (F_o_ and F are the intensities ratio in the absence and presence of PdCl_2_) and the absorbance of PdCl_2_ upon its interaction with CDs was assessed using phosphate buffer over the PH range (2–12) in two different ways. First, coordination complexes between hydroxide ions and PdCl_2_ ions can alter its quenching tendency. Second, there are many functional groups on the surface of CDs, including amine, hydroxyl, and carboxylic acid. A change in pH can change the charge of these groups, which will modify how they react to PdCl_2_ ions. The CDs and PdCl_2_ concentrations were 40 and 3.55 µg/mL respectively. It was revealed that the complex’s maximum sensitivity and absorbance was attained at pH = 7 as optimal, Fig. 3.

#### Effect of time

The absorbance and F_o_/F value of the generated complex were taken at various times in the range of 1 to 60 min in order to evaluate the impact of interaction time on stability and fluorescence quenching efficiency. After 5 min, the compound reached its maximum absorbance and quenching efficiency and remained steady for 1 h.

#### Effect of temperature

**Fig. 4:**
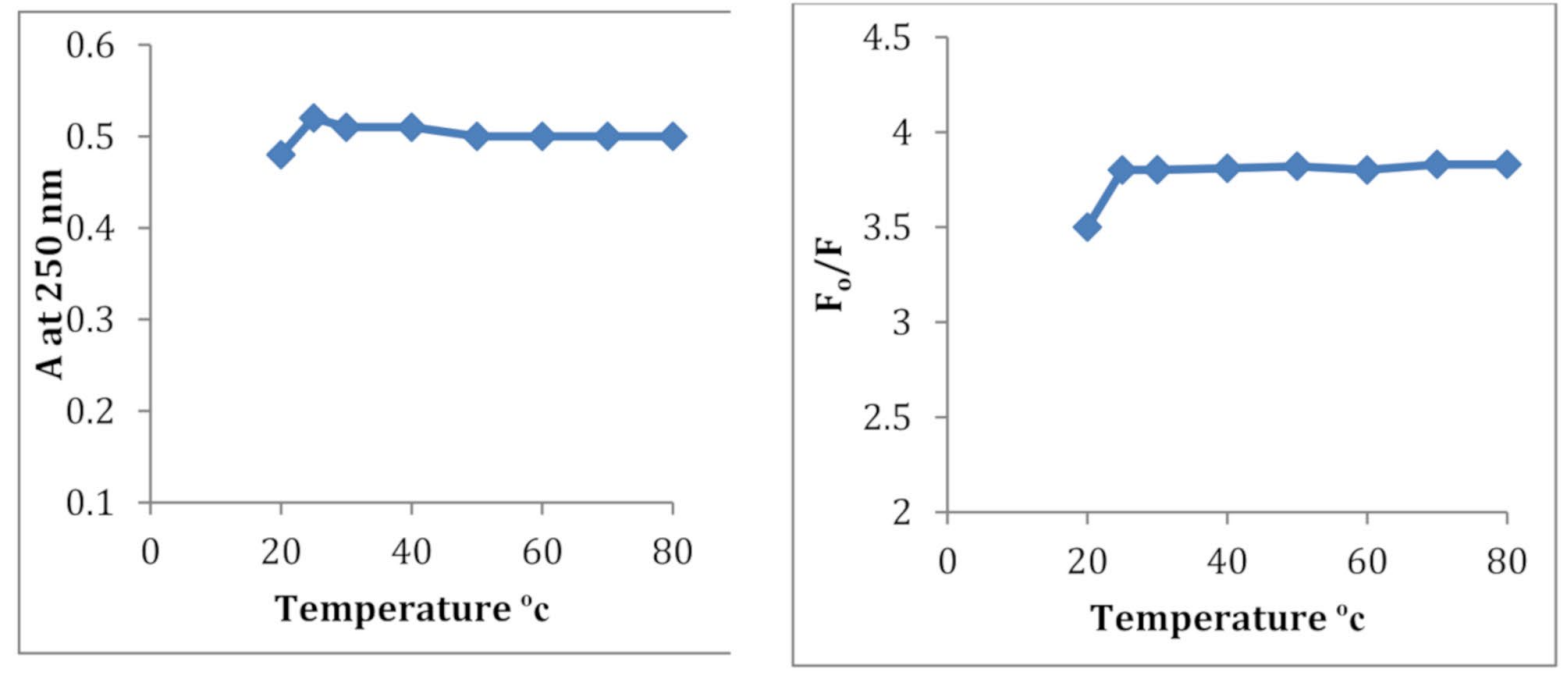
Effect of temperature on the complex formed Between PdCl_2_ and CDs reagent; PdCl_2_ = 3.55 µg/mL and 40 µg/mL CDs reagent, PH = 7

The effect of temperature on the absorbance and F_o_/F value of the generated complex was studied at range of 20–80 °C. By keeping the complex at room temperature (25 ± 2 °C) no changes were observed which indicates good sensitivity, Fig. 4.

#### Solvent selection

**Fig. 5:**
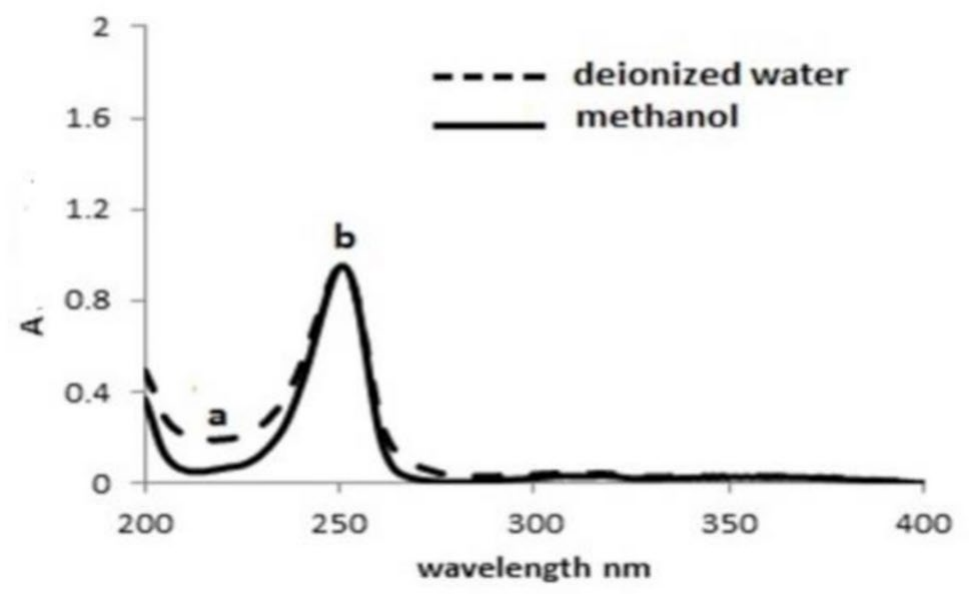
The effect of different solvents on the complex formed between PdCl_2_ and CDs reagent; PdCl_2_ = 3.55 µg/mL and 40 µg/mL CDs reagent, PH = 7

Both solvents; deionized water and methanol have been investigated using the above mentioned techniques. Satisfactory absorbance and fluorescence quenching responses were attained by deionized water that denotes good eco-friendly solvent. This offers another benefit to the proposed methods, Fig. 5.

### Analytical validation

The proposed methods were validated depending on guidelines of International Councel on Harmonization (ICH) guidelines [17].

#### Linearity

Calibration curve was constructed by plotting the concentration level of drug versus absorbance. Table 1 indicates the results for regression and statistical parameters. The linear equation reveals good correlation coefficient values (R² >0.999) with small intercepts. The high F values reflect that the mean squares are decreasing due to residuals and increasing due to regression. The small mean squares owing to residuals suggest that the experimental data points are less scattered around the regression line.

#### Detection and quantification limits

These limits were calculated according to the ICH guidelines equations [17].

For spectrophotometric methods, the DL was calculated as 3.3 σ/S and QL calculated as 10 σ/S. In these equations, σ refers to standard deviation of responses, while S refers to calibration curve’s slope. Low values of these limits indicating the sensitivity of the suggested methods, as shown in Table 1.

#### Accuracy

According to linearity ranges, three concentrations of PdCl_2_ were analyzed three times (*n* = 3). Measurement results were altered to obtain values of accepted percentage recoveries indicating high accuracy of the proposed methods Table 1.

#### Intra-day and inter-day precision

The precision was estimated as the relative standard deviations of three concentrations of PdCl_2_ measured three times each on the same day (intra-day). A procedure similar to this was used to calculate the inter-day precision but on three days. The collected data indicate that the RSD% is less than 2%. This means the high level of precision of the suggested methods Table 1.

**Table 1:** Assay parameters for the determination of PdCl_2_ by the proposed methods

Parameters	Spectrophotometric method	Fluorimetric method	
		F_o_-F	F_o_/F
$\:{{\lambda\:}}_{nm}$ Conc. Range (µg/mL)	250 nm	λem 432	
	0.71–3.55	0.0088–0.8870	0.0088–0.8870
a^**1**^	0.236	191.105	1.0
b^**2**^	0.0941	15.105	43.71
r^3^	0.9999	0.9995	0.9999
$\:{S}_{a}$	0.00049	0.0870	0.0035
$\:{S}_{b}$	0.00025	0.1619	0.0065
$\:{S}_{b}^{2}$	6.25 Х$\:{10}^{-8}$	0.0262	4.225Х$\:{10}^{-5}$
$\:{S}_{b}\%$	0.267	1.072	0.015
LOD ( µg/mL)	0.0173	0.0190	0.00026
LOQ ( µg/mL)	0.0526	0.0576	0.00081
Significance F	4.211	7.924	3.109
**Accuracy** (mean ± RSD%)	100.14 ± 0.46	99.28 ± 0.31	99.94 ± 0.22
**Precision (RSD%)**			
(Intraday precision)	0.65	0.45	0.30
(Inter day precision)	0.28	0.16	0.15

^1^ The intercept

^2^ The slope

^3^ The regression coefficient

### Method comparison

According to our suggested procedures, assay of PdCl_2_ is performed. Each method’s specifications are analyzed using the chosen wavelength and appropriate fluorescence intensity. As demonstrated in Table 2, the obtained recovery% and RSD% values verify the precision and accuracy of these procedures. The variance ratio F-test and the Student’s t test are effective tools for comparing our two suggested procedures statistically [18]. The comparison’s results reveal that there is no discernible difference between the procedures in terms of accuracy or precision Table 2.

**Table 2:** Application of the proposed methods for the determination of PdCl_2_

Proposed methods	Spectrophotometric method	Fluorimetric method (F_o_/F)
Recovery %^1^ ± % RSD^2^	100.08 ± 0.28	99.94 ± 0.22
t^3^	0.14	
F^4^	3.27	

^1^ Average of three determinations

^2^Relative standard deviation

^3^ Represents calculated values of t

^4^ Represents calculated values of F

### Application on pharmaceutical Preparation (Naprofen^®^)

In order to further demonstrate the applicability of the proposed methods, a sample of Naprofen^®^ tablet that spiked with 0.002 mM PdCl_2_ was analyzed. The obtained results showed % recovery and %RSD values ranging from 98.61 ± 1.30 and 99.30 ± 0.89 for spectrophotometric and fluorimetric methods, respectively. These indicate the applicability of the developed methods for the determination of PdCl_2_ with good levels of accuracy, precision and selectivity.

### Greenness assessment

**Fig. 6:**
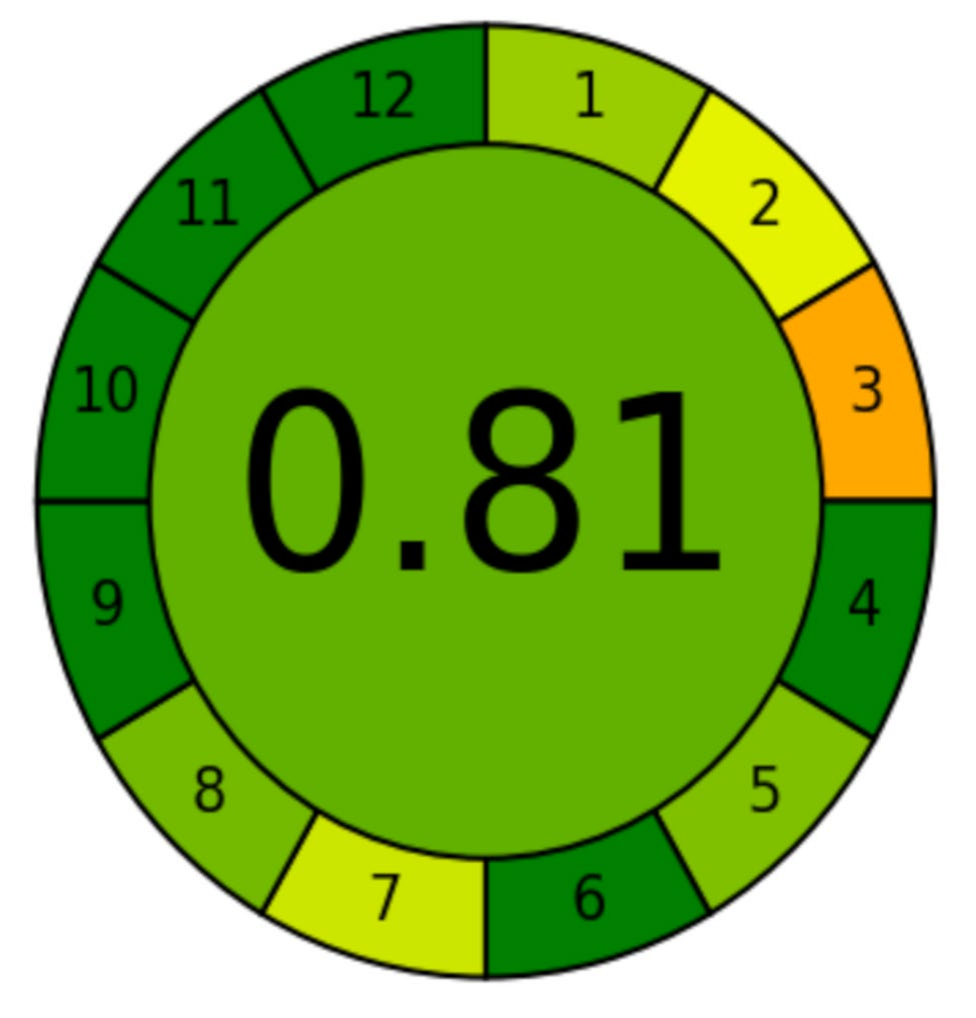
AGREE profile of the proposed assay method

Environmental impact on the analytical method is directly assessed using two greenness matrices; AGREE and Complex-GAPI. The present study involves AGREE which is countable measure illustrated as round-shaped pictogram of 12 components of Green Analytical Chemistry (GAC). As the resulting score ranges from 0 to 1, it turned to be 0.81, Fig. 6. The latter value is an absolute proof for greenness clarification and relatively high environmental satisfaction [19].

**Fig. 7:**
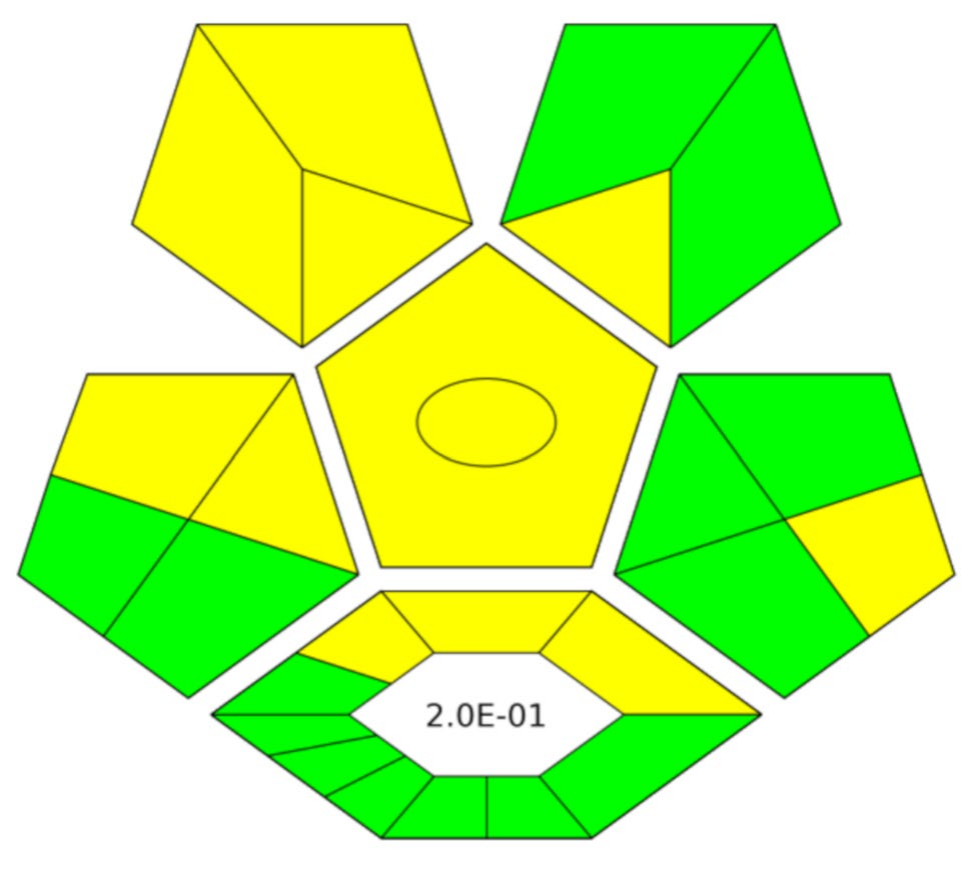
Six-block regular pictogram produced by Complex-GAPI tool for greenness assessment of the proposed fluorimetric study

Complex-GAPI is a trend of GAPI tool; assessing, in addition to the analytical procedure itself, the sample preparation steps. This tool is a best fit for our present study that likely involves, the use of natural precursor for carbon dots, as potent fluorophore. As shown in Fig. 7, the dominant colors are the green and yellow ones; illustrating the environmental friendship and lack of any harmful components. In numerical conclusion comes the E-factor, which is defined as the ratio between the total mass of process waste and the total mass product. The lower the E-factor, the more ecological safety of the method [20].

## Conclusions

A green and economic synthesis strategy for compatible fluorescent water soluble carbon dots (CDs) was reported. This probe is utilized to selectively, sensitively and reproducibly detect PdCl_2_ as impurities in Naproxen drug. These benefits permit using the developed techniques in routine analysis for Pd(II) examination in diverse objects. Additionally, the development of CDs based optical sensors may combine this potent and intriguing application to sensing, opening up new possibilities for sensor design and the highly sensitive and selective detection of a variety of analytes.

## Supplementary Information

Below is the link to the electronic supplementary material.

Supplementary Material 1

## Data Availability

The datasets used and/or analyzed during the current study are available from the corresponding author on reasonable request.
